# 

**Published:** 1896-04

**Authors:** 


### Communicated.

### ILLINOIS STATE DENTAL SOCIETY.

The thirty-second annual meeting of the Illinois State Dental Society will be held in the Senate Chamber, Springfield, Ill., May 12 to 15, 1896. The executive committee has been especially fortunate in preparing a very interesting programme. No member can afford to be absent. Dentists practicing in the State are cordially invited to attend and if possible to become members of the Society. The profession outside of the state is always welcome at these meetings. The hotels and railroads have granted the usual reduction. Pay full fare in coming and take receipt therefor, this when countersigned by the Secretary, entitles the holder to return at one-third the usual fare.

LOUIS OTTOFY, Secretary,
Masonic Temple, Chicago.

### To Readers and Correspondents.

Communications intended for publication, and exchange journals and papers, should be addressed to the Editor of The American Journal of Dental Science, No. 845 N. Eutaw St. Baltimore, Md. All letters relating to the business of the Journal, or containing cash remittances, to be directed to Snowden & Cowman Mfg. Co., 9 W. Fayette Street, Baltimore, Md. Articles for publication should be received by the Editor at least two weeks previous to the first of the month on which the number in which it is designed for them to appear is issued.

☞ The American Journal of Dental Science will contain fifty pages of reading matter in each number, making a volume of about Six Hundred pages,

EXCLUSIVE OF ADVERTISEMENTS.

